# Sorafenib exerts an anti-keloid activity by antagonizing TGF-β/Smad and MAPK/ERK signaling pathways

**DOI:** 10.1007/s00109-016-1430-3

**Published:** 2016-06-24

**Authors:** Wenbo Wang, Miao Qu, Lan Xu, Xiaoli Wu, Zhen Gao, Tingyu Gu, Wenjie Zhang, Xiaoyan Ding, Wei Liu, Yue-Lei Chen

**Affiliations:** 1 Department of Plastic and Reconstructive Surgery, Shanghai Ninth People’s Hospital, Shanghai Jiao Tong University School of Medicine, Shanghai Key Laboratory of Tissue Engineering, 639 Zhi Zao Ju Road, Shanghai, 200011 People’s Republic of China; 2 Stem Cell Bank/Stem Cell Core Facility, Institute of Biochemistry and Cell Biology, Shanghai Institutes for Biological Sciences, Chinese Academy of Sciences, 320 Yueyang Road, Shanghai, 200031 People’s Republic of China; 3 National Tissue Engineering Center of China, Shanghai, People’s Republic of China

**Keywords:** Sorafenib, Keloids, Keloid fibroblast, TGF-β/Smad signaling, MAPK/ERK signaling

## Abstract

**Abstract:**

Keloid disease is characterized by hyperproliferation of responsive fibroblasts with vigorously continuous synthesis of extracellular matrix (ECM) components. Although the process by which keloids develop is poorly understood, most theories of the etiology are referred to fibroblast dysfunction. A central event in dermal repair is the release of growth factors in response to skin injury, which leads to the dysregulation of several crucial pathways that initiate the activation of keloid fibroblasts (KFs) and promote ECM accumulation. Hence, strategies aimed at reducing the production of these cytokines and/or disrupting their intracellular signal transduction have potential clinical significance for curing keloid. As the first oral multikinase inhibitor, sorafenib blocks a number of intracellular signaling pathways which are also pivotal for keloid pathogenesis. Therefore, evaluation of the effects of sorafenib on keloid disease seems timely and pertinent. In this study, we reported the identification of sorafenib that antagonized TGF-β/Smad and MAPK/ERK signaling pathways in primary KFs. Impressively, treatment with sorafenib inhibited KF cell proliferation, migration, and invasion, and simultaneously reduced collagen production in KFs. Furthermore, we present ex vivo evidence that sorafenib induced the arrest of KF migration, the inhibition of angiogenesis, and the reduction of collagen accumulation. These preclinical observations suggest that sorafenib deserves systematic exploration as a candidate agent for the future treatment of keloids.

**Key message:**

- The intracellular TGF-β/Smad and MAPK/ERK signaling pathways is blocked by sorafenib.
- Sorafenib inhibits the proliferation, migration, invasion, and ECM deposition in keloid fibroblasts.
- Sorafenib reduces KF migration and concomitantly angiogenesis in keloid explants.
- Sorafenib is a promising agent for the treatment of keloids and hypertrophic scars.

**Electronic supplementary material:**

The online version of this article (doi:10.1007/s00109-016-1430-3) contains supplementary material, which is available to authorized users.

## Introduction

As an abnormal process of wound healing, keloids appear as firm, rubbery lesions or glossy, fibrous nodules. Unique to humans, keloids are characterized by an uncontrolled growth of dermal fibroblasts with an excessive production of the extracellular matrix (ECM), especially collagen types I (late), III (early), fibronectin, and other ECM proteins such as elastin and periostin [1, 2]. The prevalence of keloids and the risk of recurrence are relatively high in people of Asian or African descent with a genetic predisposition [3, 4], which have plagued physicians and patients for centuries. Although multiple localized therapeutic modalities exist, including surgical excision, cryotherapy, topical drug injection, and radiotherapy [5, 6], the clinical results remain unsatisfactory. Simultaneously, the adverse effects and inconvenience of the current treatments result in poor patient compliance, signifying that the treatment of keloids is a thorny issue. Hence, research efforts on the pathogenetic mechanisms underlying keloid formation and development are imperative for curing this disease.

The molecular basis of keloids is complicated and involves both genetic and environmental factors, such as gene mutations, aberrant cytokine expression, inflammatory responses, and UV exposure [3, 7]. Although the etiology of keloids remains largely unknown, the histopathological hallmarks of this disease are referred to fibroblast dysfunction. Primary fibroblasts isolated from human keloids exhibit a typical spindle morphology that is similar to the size and shape of normal dermal fibroblasts, but they possess tumor-like properties with a great capacity for cell proliferation, migration, and high production of collagens and certain growth factors [1, 8]. Among these mediators, transforming growth factor-β (TGF-β) is believed to be a master inducer of keloid development [9]. Of note, the expression levels of biologically active isoforms of TGF-β ligands and their receptors are markedly elevated in keloid fibroblasts (KFs) compared with normal dermal fibroblasts [10, 11]. Furthermore, TGF-β is vital for cell proliferation and collagen synthesis in KFs, whereas KFs display a distinct sensitivity to TGF-β stimulation [12, 13]. In addition to TGF-β signaling, a multitude of cytokines have been reported to be dysregulated in keloid pathogenesis and recurrence, such as vascular endothelial growth factor (VEGF) and platelet-derived growth factor (PDGF) [1, 9]. These secreted molecules regulate a broad array of intracellular responses in keloids including ECM production and deposition, fibroblast proliferation, angiogenesis, and inflammatory cell infiltration. Therefore, attempts to reduce the synthesis of selected cytokines or block their associated signal transduction may provide an alternative potential approach for keloid treatment.

Over the past decades, the discovery and development of small molecules that target multiple signaling pathways have revolutionized the fight against cancer. Most notably, sorafenib (trade name: Nexavar) is the first oral multikinase inhibitor that was approved by the Food and Drug Administration (FDA) for the treatment of patients with a broad range of tumor types [14, 15]. As a potent tyrosine kinase inhibitor (TKI), sorafenib blocks tumor angiogenesis and progression by targeting Raf kinase and several receptor tyrosine kinases, including VEGF receptor 2 (VEGFR2) and PDGF receptor β (PDGFRβ) [16]. Histologically, keloids are benign fibrotic tumors of the dermis characterized by a collection of atypical fibroblasts with overexpression of cytokines and increased angiogenesis [8, 17]. Therefore, evaluation of the effects of sorafenib on keloid disease seems timely and pertinent. Previously, we reported the potential role of sorafenib in the inhibition of epithelial-mesenchymal transition in hepatocytes [18]. Here, we demonstrated that sorafenib not only reduced the proliferation, invasion, and ECM production of KFs in vitro but also inhibited KF migration, angiogenesis, and collagen accumulation in cultured keloid explants ex vivo, suggesting an attractive therapeutic potential of this drug for treating skin scars and keloids.

## Material and methods

### Patients, tissue samples, and chemical reagents

Thirty-six keloid scar specimens in the active stage were harvested from 30 patients (13 men and 17 women, age range 20–55 years; details listed in Supplementary Table S1), who did not receive any treatment for their keloids before surgical excision. All the samples were exclusively derived from chest keloids. To minimize individual variation, human fibroblasts derived from three keloid specimens from different patients were mixed as a cell pool. Protocols for the handling of human tissues and cells were approved by the Ethics Committee of Shanghai Jiao Tong University School of Medicine, and all tissue samples were donated by the patients for research purposes with written informed consent.

Sorafenib (Bayer Pharmaceuticals) was dissolved in 100 % DMSO to a stock concentration of 10 mM and stored under light-protected conditions at −20 °C. On the day of use, sorafenib was diluted directly to the desired dose with an identical final concentration of DMSO. The final concentration of DMSO did not exceed 0.2 % (*v*/*v*) in any experiment described below, which was nontoxic to KFs (Supplementary Fig. S1).

## Isolation and culture of normal and keloid fibroblasts

The chest keloid tissues were excised under local anesthesia and collected in a 50-ml centrifuge tube, followed by washing three times in 2.5 % chloramphenicol solution for 5 min each time. The tissues were soaked three times in phosphate-buffered saline (PBS) for 5 min each time. Afterward, the epidermis was removed using a scalpel, and the remaining dermis was minced into small pieces followed by enzyme digestion with 0.3 % collagenase II dissolved in Dulbecco’s modified Eagle’s medium (DMEM) for 4 h at 37 °C on a rotator. After digestion, the collected cells were centrifuged at 1500 rpm for 5 min and then resuspended in DMEM supplemented with 10 % fetal bovine serum (FBS) and penicillin/streptomycin. The cells were seeded onto a 10-cm culture dish at a density of 2 × 10^4^/cm^2^ and cultured in a humidified 5 % CO_2_ atmosphere at 37 °C. When the cells reached 80 % confluence, they were detached with 0.25 % trypsin-EDTA and subcultured at the same density. The primary and first passage cells were used in the following experiments. Human foreskin fibroblasts were isolated from nine normal individuals (age range 1–10 years) using the procedure as described previously [19].

## CCK-8 cell proliferation assay

The primary KFs were seeded in 96-well plates with 1000 cells per well along with 200 μl of medium. After being starved in serum-free medium for 24 h to allow for cell synchronization, the KFs were replaced with fresh culture medium in the presence or absence of sorafenib (5 μM) and then tested with a cell counting kit-8 (CCK-8; Dojindo, Japan) at days 2, 4, 6, and 8. Briefly, at each testing time point, 10 μl of sterile CCK-8 solution was added to each well and incubated for 2 h at 37 °C, and then the medium was harvested for measuring the optical density values at 450 nm using a microplate reader (Thermo Scientific). All the assays were performed in quintuplicate and repeated using three cell samples.

## Cell cycle analysis

To perform the test, KFs were cultured without or with sorafenib at a concentration of 5 μM for 2 and 6 days, respectively. The cells were then trypsinized, centrifuged at 3000 rpm for 10 min, and stained in ice-cold PBS solution containing 100 μg/ml propidium iodide (PI), 0.1 % Triton X-100, 0.1 % sodium citrate, and RNase (1 mg/ml). The samples were incubated at 4 °C overnight, and flow cytometric analyses were performed using a flow cytometer (Beckman Coulter) equipped with ModiFit LT v2.0 software. The analyses were performed on days 2 and 6 and repeated in four cell samples.

## EdU incorporation assay

After being treated with or without 5-μM sorafenib for 48 h, the KFs were exposed to 5-ethynyl-20-deoxyuridine (EdU at a working concentration of 10 μM) in culture medium for additional 3 h. EdU incorporation was detected at room temperature using the Click-iT™ EdU Alexa Fluor Imaging kit (Invitrogen/Molecular Probes) according to the user’s manual. The cells were fixed in 3.7 % formaldehyde for 15 min at room temperature, permeabilized by incubation with 0.5 % Triton X-100 in PBS for 20 min, and then stained with the Click-iT™ reaction cocktail for 30 min, followed by staining with Hoechst 33342 dye for 30 min. The EdU-positive cells (red) and Hoechst-positive cells (blue) were imaged under a fluorescent microscope (Olympus, Japan) and counted using Image-Pro Plus (IPP) 6.0 software (Media Cybernetics). The incorporation ratio of EdU-positive cells was shown as the percentage of EdU-positive cells of total Hoechst-positive cells. The results were counted in five randomly selected fields and repeated in three independent experiments.

## RNA isolation and real-time quantitative PCR

Total RNA isolation and reverse transcription were performed essentially as previously described [20]. Quantitative PCR (qPCR) was performed using a Power SYBR Green PCR master mix (Applied Biosystems) in a real-time thermal cycler (Stratagene). The amplified complementary DNA (cDNA) products were normalized with the *hypoxanthine phosphoribosyltransferase 1* (*HPRT1*) cDNA content. The primers for real-time qPCR analysis are listed in Supplementary Table S2. Each assay was performed in triplicate and repeated in three cell samples.

## Cell migration assay

A transwell system and an in vitro scratch wound assay were used to evaluate cell migration. For the in vitro wound assay, when reaching 90 % confluence in 6-well plates, the KFs were treated with 10 μg/ml mitomycin C (Sigma) for 2 h and washed with basal media. The cell monolayer was scratched with a sterile 200-μl pipette tip and incubated in the culture medium with or without sorafenib (5 μM) for 24 h. Photographs were taken after scratching and at the indicated time point. The photographed area was quantified with computer-assisted image analysis using IPP 6.0 software. The results are presented as a percentage of the area of the scratch filled by the KFs. The data were acquired from five randomly selected high-power fields.

A transwell system (Merck Millipore, 8-μm pore size) was used for the assay. Briefly, KFs were starved for 12 h in serum-free DMEM. Then, the cells were harvested and seeded in the upper compartment of the chamber in 24-well plates using a total of 1 × 10^4^ cells in 0.1 ml of serum-free DMEM with or without sorafenib (5 μM), and the bottom well was filled with 0.6 ml of DMEM plus 10 % FBS. The cells were incubated for 24 h. Afterward, a cotton swab was used to remove the nonmigrated cells on the upper surface of the membrane, and the migrated cells on the bottom surface of the filter membrane were fixed in 4 % paraformaldehyde and stained with DAPI. The numbers of the migrated cells were obtained by counting the stained cells in five randomly selected fields under the microscope.

## Cell invasion assay

A cell invasion assay was conducted as described previously [21]. Briefly, KFs in a total of 5 × 10^4^ cells were seeded into the upper compartment of the chamber coated with Matrigel (BD Biosciences) along with DMEM containing 2 % FBS with or without sorafenib (5 μM) in a total volume of 0.1 ml, and the lower chamber was filled with DMEM plus 10 % FBS in a total volume of 0.6 ml. After 24 h of culture, the nonmigrated cells on the upper surface of the membrane were removed with a cotton swab, and the migrated cells that crossed the Matrigel barrier on the bottom surface of the membrane were fixed, stained with DAPI, and counted in five random fields. The mean value for each chamber was determined from three independent experiments.

## Luciferase assay

KFs in a 24-well plate were co-transfected with (CAGA)_12_-Lux reporter (200 ng per well) and pRL-SV40 plasmid (5 ng per well). After transfection for 24 h, the cells were incubated in serum-free medium in the absence or presence of sorafenib for an additional 12 h prior to harvesting. Luciferase activity was detected using a Dual Luciferase Reporter Assay System (Promega) according to the manufacturer’s instructions.

## ELISA assay

After 18 h of incubation with or without sorafenib (5 μM) in serum-free culture medium, the released TGF-β1 protein from KFs was measured using an ELISA Development Kit (Anogen) according to the manufacturer’s instructions with an absorbance measurement at a wavelength of 450 nm. Similarly, the concentration of VEGF in the supernatant was determined by a human VEGF ELISA kit (ExCell Bio). All the assays were performed in triplicate and repeated in three cell samples.

## Western blotting analysis

After 4 h of incubation in the absence or presence of sorafenib as indicated, total protein was extracted with RIPA lysis buffer as described previously [22]. The prepared protein was subjected to SDS-PAGE and subsequently transferred onto PVDF membranes. The membranes were incubated with primary antibodies as indicated in the figures, followed by incubation with appropriate HRP-conjugated secondary antibodies (Jackson ImmunoResearch). The protein bands were eventually visualized using an enhanced chemiluminescence (ECL) detection kit (Amersham). The primary antibodies against Phospho-Erk1/2 (Thr^202^/Tyr^204^), Erk1/2, Phospho-p38 MAPK (Thr^180^/Tyr^182^), p38 MAPK, Phospho-SAPK/JNK (Thr^183^/Tyr^185^), SAPK/JNK, Phospho-Smad2 (Ser^465/467^), and Smad4 were purchased from Cell Signaling Technology. The rest include antibodies against β-actin (Sigma-Aldrich), Smad2/3 (BD Biosciences), Phospho-Smad3 (Ser^423/425^; Invitrogen), and Smad7 (Santa Cruz).

## Ex vivo explant culture of human keloid tissue

After the harvest of excised keloid tissue under aseptic conditions, the epidermis was removed, and the left dermis was cut into 3 mm × 2 mm × 2 mm small pieces using a scalpel. The keloid dermal fragments were divided into three groups and seeded onto 10-cm culture dishes for 3 days in 6 ml of DMEM containing 10 % FBS as described previously [23, 24]. After tissue attachment, the medium was replaced with different doses of sorafenib (0, 5, and 20 μM). Representative micrographs were obtained in the same location where the KFs were scratched from at the different time points on days 2, 4, and 7. After treatment with sorafenib for 7 days, the migrated KFs were collected and counted using a hematocytometer.

## Histological and immunohistochemical analyses

The keloid specimens were fixed in 4 % paraformaldehyde at 4 °C overnight, embedded in paraffin blocks, and sectioned to 5-μm thicknesses. The sections were stained with hematoxylin and eosin (H&E) for routine examination. In addition, the keloid sections were incubated with antibodies against Phospho-Smad2/Smad3, Phospho-Erk1/2, CD31, CD34, collagen I, and collagen III at a dilution of 1:500. The bound antibodies were eventually visualized using 3,3′-diaminobenzidine (DAB) as a chromogen (Dako), and the slides were counterstained with hematoxylin. The numbers of CD31^+^ and CD34^+^ vessels were obtained in six randomly selected fields under the microscope.

## Statistical analysis

All data are presented as the mean ± standard deviation (SD), and the statistical analyses were analyzed using the statistical software Statistical Package for the Social Sciences (SPSS version 19.0). A Student’s *t* test was applied to analyze the difference between the control and sorafenib-treated groups. *p* < 0.05 was considered statistically significant.

## Results

### Elevated levels of phosphorylated forms of Smad2/Smad3 and Erk-1/2 in keloids

**Fig. 1:**
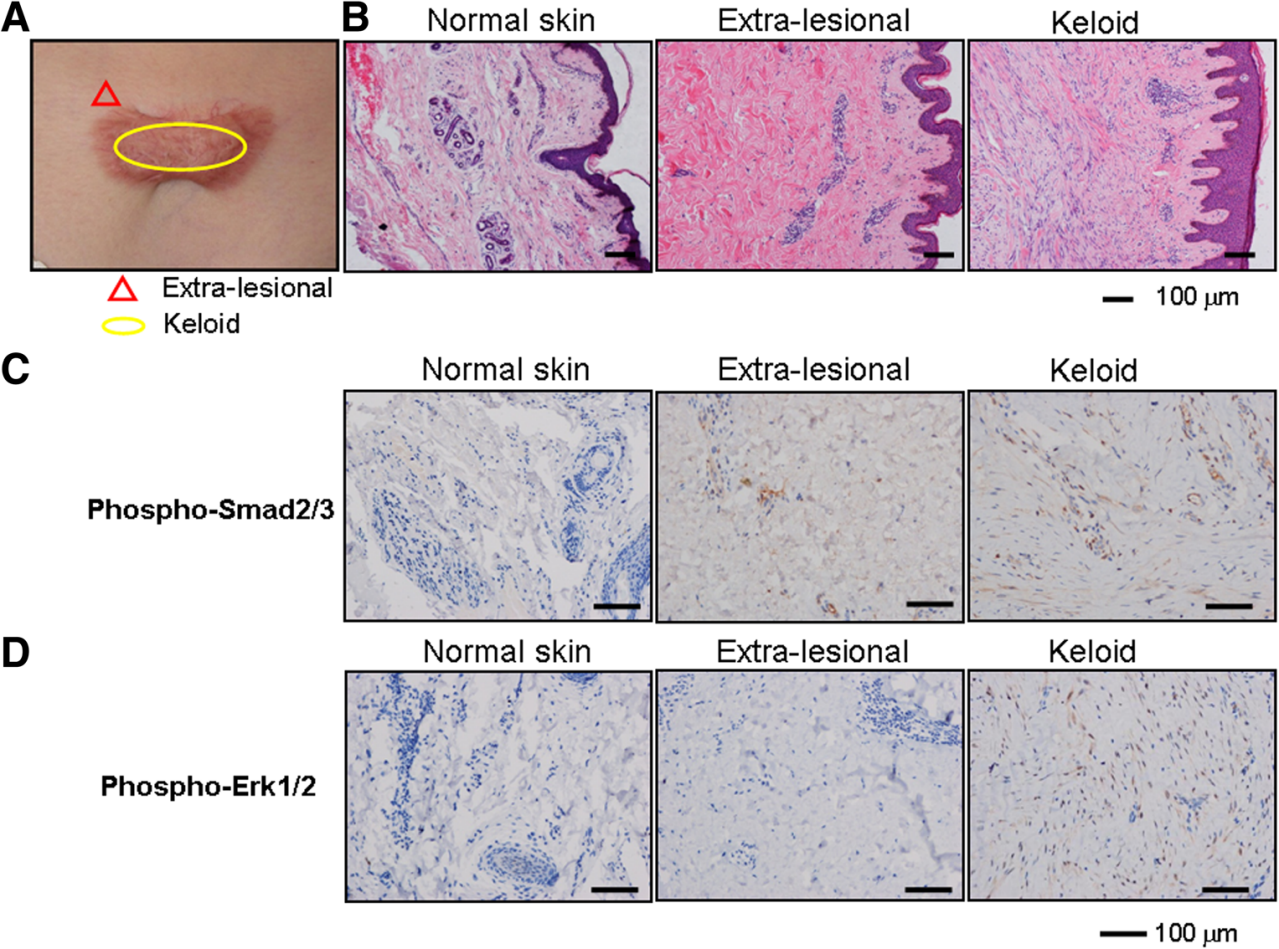
The aberrant activation of TGF-β/Smad and MAPK/ERK signaling pathways in keloids. **a** Schematic representation of a keloid and its extra-lesional tissue samples (the sample harvested from the same patient as an internal control). **b** Histological analysis of normal skin, keloid, and its extra-lesional tissue samples using H&E staining. **c**, **d** Immunohistochemical staining using antibodies against Phospho-Smad2/3 and Phospho-Erk1/2

Keloid disease is a fibroproliferative lesion that forms in the dermis during a prolonged wound healing period [25]. The most striking feature that distinguishes normal skin from human keloid samples is the presence of an undulating dermal-epidermal junction and skin appendages in the dermis (i.e., hair follicles, sweat glands, and sebaceous glands), with the keloid epidermis being thickened with flattened rete ridges and dermal papillae (Fig. 1a, b). Histopathological analysis also revealed that numerous thick bundles of collagenous matrices were arranged in a reticular manner to form a characteristic whorled pattern (Fig. 1b).

Numerous studies have recognized that TGF-β signaling plays a vital role in fibropathogenesis [26, 27]. Hence, we first detected whether TGF-β signaling was upregulated in human keloid samples. Indeed, immunohistochemical analysis in the keloid sections showed intense phosphorylated forms of Smad2/Smad3 (Fig. 1c), which are central mediators of intracellular TGF-β signal transduction [28]. Meanwhile, the phosphorylation of p44/42 MAPK (Erk1/2) was enhanced in human keloids but not normal skin (Fig. 1d). These observations suggested that both TGF-β/Smad and MAPK/ERK signaling pathways were highly activated in human keloids, making them potential pharmacological targets for the clinical treatments of keloids.

## Sorafenib blocked intracellular signaling cascades in KFs

**Fig. 2:**
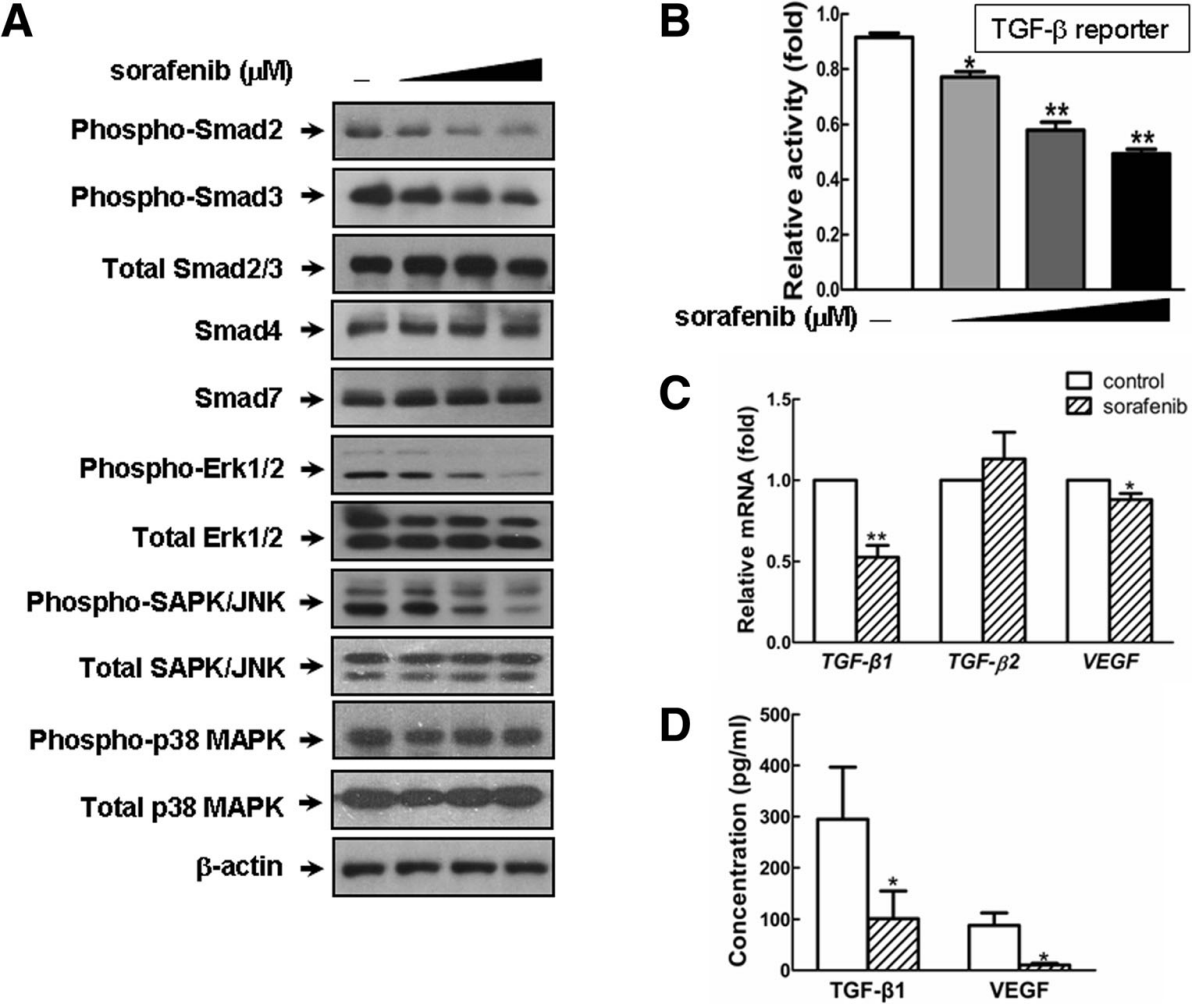
Sorafenib antagonizes intracellular signaling in vitro. **a** KFs were treated with increasing doses of sorafenib (0, 2.5, 5, and 10 μM) for 4 h and harvested for western blot (WB) analysis to assess the intracellular signaling as indicated. **b** As described in the “Material and Methods” section, KFs seeded in 24-well plates were transfected with (CAGA)_12_-Lux reporter, incubated with increasing doses of sorafenib (0, 2.5, 5, and 10 μM) for 8 h and then analyzed with a luciferase assay. **c** After treatment with sorafenib (5 μM) for 24 h, the KFs were subjected to real-time qPCR to detect the gene expression levels of two major profibrotic members of the TGF-β superfamily (*TGF-β1* and *TGF-β2*) and *VEGF*. **d** The concentrations of secreted TGF-β1 and VEGF in the supernatant were determined by ELISA after 18 h of sorafenib treatment (5 μM). Each assay was performed in triplicate and repeated in four independent cell pools (*n* = 4, 12 samples). The *asterisks* denote significant differences (*p* < 0.05) within experiments, as determined by the Student’s *t* test

The findings above prompted us to seek small chemicals that target both TGF-β/Smad and MAPK/ERK signaling pathways. As the first FDA-approved multikinase inhibitor in cancer therapy, sorafenib is known to target a couple of receptor tyrosine kinases [16]. To evaluate the efficacy of sorafenib on the intracellular signal transduction that is involved in keloid pathogenesis, we isolated primary fibroblasts from human keloid samples, which have been widely used as an in vitro model to study wound healing and drug metabolism [29, 30]. As shown in Fig. 2a, sorafenib evidently abrogated the phosphorylation of Smad2 and Smad3 at a workable concentration of 5 μM, whereas the expression of Smad4 and Smad7 was unaffected. Simultaneously, sorafenib reduced the phosphorylated forms of p44/42 MAPK (Erk1/2) as well as SAPK/JNK, but not p38 MAPK (Fig. 2a). Given the vital role of TGF-β signaling in KF activation after wounding [9, 13], we then detected the impact of sorafenib on this signaling using (CAGA)_12_-Lux, a luciferase reporter that contains 12 copies of the Smad-binding element [31]. Notably, this reporter was capable of being inhibited by sorafenib in a dose-dependent manner (Fig. 2b). Moreover, sorafenib resulted in an evident decrease of *TGF-β1* and *VEGF* transcripts and extracellular secretion, indicating that both the mRNA and protein levels of TGF-β1 and VEGF were inhibited after sorafenib treatment (Fig. 2c, d). Taken together, these data revealed that sorafenib acts as an effective inhibitor of TGF-β/Smad and MAPK/ERK signaling cascades in vitro.

## Sorafenib inhibited cell proliferation and the cell cycle in KFs

**Fig. 3:**
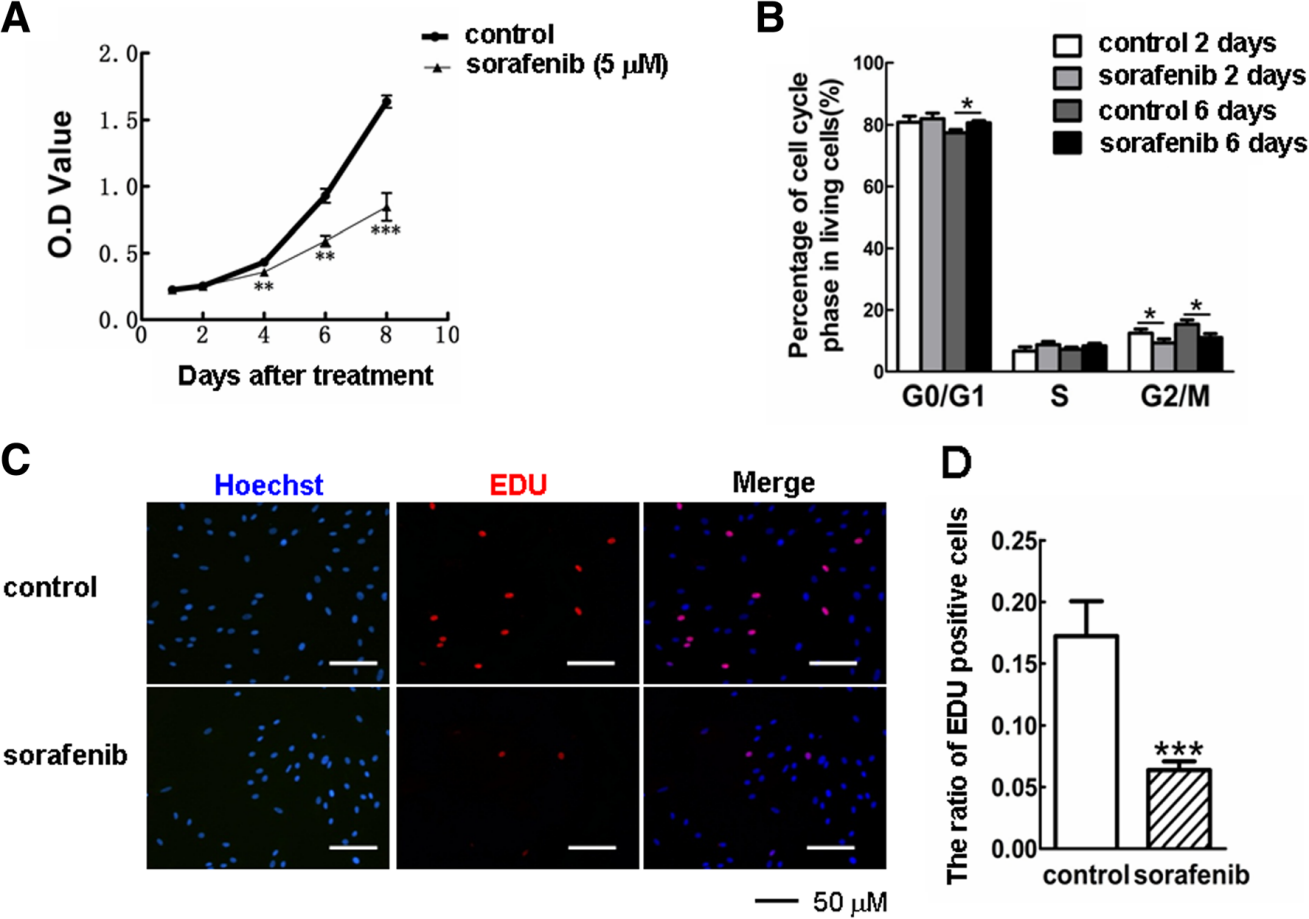
Treatment with sorafenib inhibits KF proliferation. **a** The cell viabilities of KFs treated with or without sorafenib (5 μM) were measured with a CCK-8 assay at days 2, 4, 6, and 8. **b** The effect of sorafenib (5 μM) on the cell cycle profiles was further evaluated by flow cytometry analysis. **c** After treatment with sorafenib (5 μM) for 96 h, KFs were incubated with EdU (10 μM) for an additional 3 h. The samples were imaged under a fluorescent microscope. **d** The ratio of EdU-positive cells to Hoechst-labeled cells in each group was determined. All the experiments were repeated in three independent cell pools (*n* = 3, 9 samples). *G0/G1* gap between end of M-phase and start of S-phase, *S* DNA duplication phase, *G2* gap between end of S-phase and start of M-phase, *M* mitosis

Due to the aberrant proliferation of dermal fibroblasts that have been shown to contribute greatly to keloid overgrowth and expansion [8, 32], we then investigated the ability of sorafenib to modulate the proliferation of KFs. As determined by CCK-8, a colorimetric assay used to measure cell viability and cytotoxicity, sorafenib significantly inhibited the proliferation of KFs in a time-dependent manner compared with the nontreated group (Fig. 3a). Additionally, the exposure of KFs to sorafenib for 2 and 6 days eventually led to a decrease of cells in the G2/M phase and an increase of cells in G0/G1 at day 6 (Fig. 3b). No significant difference was found in the sub-G1 population between the control and sorafenib-treated groups at both days 2 and 6 (*p* > 0.05; data not shown).

Next, we delineated the inhibitory effects of sorafenib on cell growth using an EdU incorporation assay. As shown in Fig. 3c, the DNA synthesis in KFs was rapidly decreased by sorafenib as fewer KFs were found to be able to incorporate EdU compared with the cell number of nontreated group. Quantitative analysis revealed a noticeable difference in the percentage of EdU-positive cells between the control and sorafenib-treated groups (*p* < 0.001, Fig. 3d), indicating that sorafenib is likely to inhibit cell proliferation via cell cycle arrest.

## Sorafenib attenuated collagen production and ECM accumulation in KFs

**Fig. 4:**
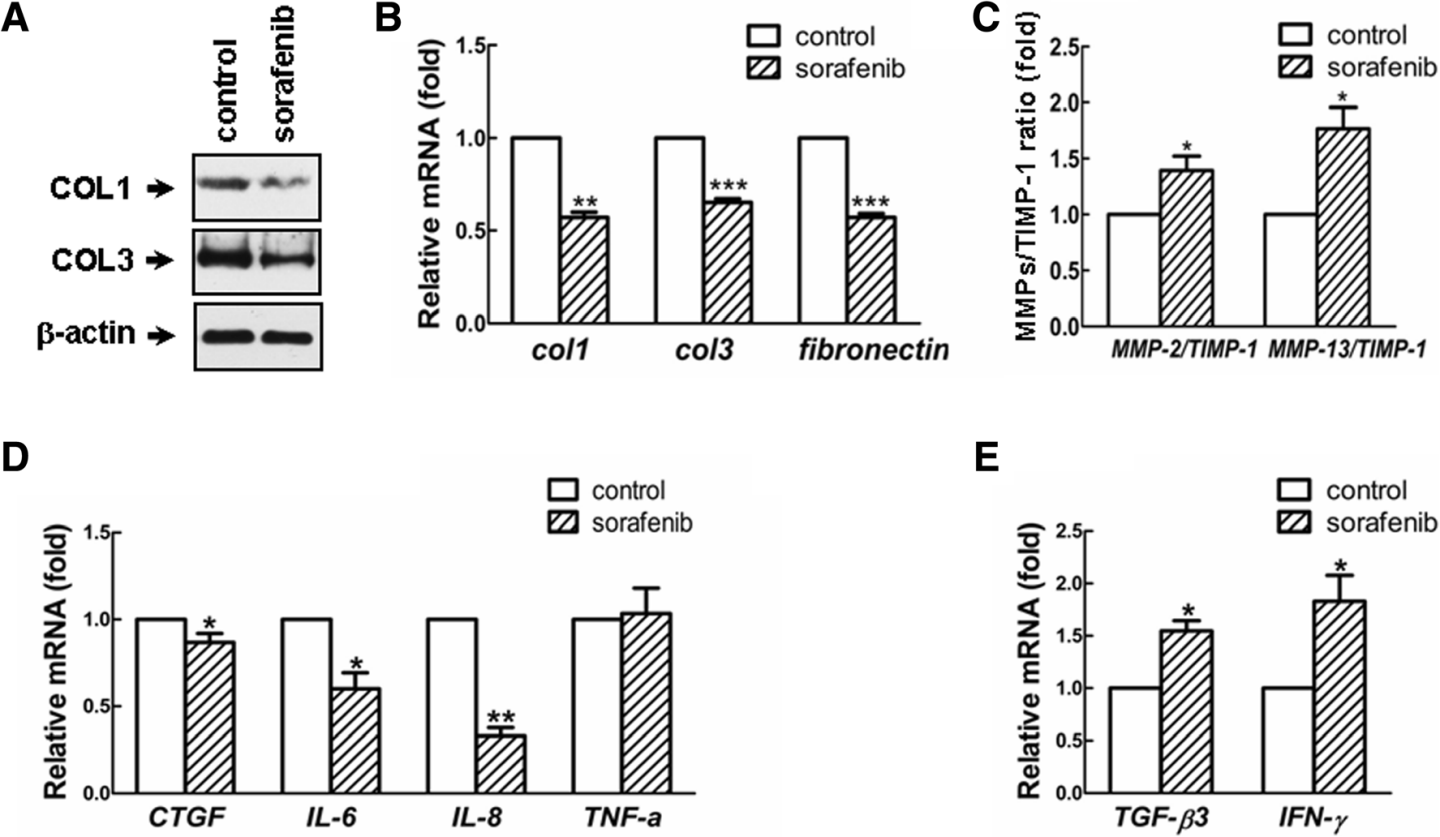
The antifibrotic role of sorafenib in counteracting ECM production and accumulation. After treatment with sorafenib (5 μM) for 48 h, KFs were subjected to Western blotting to determine the effects of sorafenib on the collagen accumulation (**a**). Similarly, real-time qPCR was performed to detect the roles of sorafenib on the gene expression levels of ECM molecules (**b**), the ratio of *MMPs/TIMP-1* (**c**), pro-fibrotic genes (**d**), and antifibrotic genes (**e**). Each assay was performed in triplicate and repeated in three independent cell pools (*n* = 3, 9 samples)

Afterward, we explored whether sorafenib treatment could reduce the endogenous expression of collagen and other ECM molecules in primary KFs. Impressively, the exposure of KFs to sorafenib for 48 h remarkably inhibited both transcriptional and translational levels of fibrotic matrix components, such as types I and III collagens (COL1 and COL3) (Fig. 4a, b), implying a protective role for sorafenib in counteracting collagen production and accumulation. Likewise, sorafenib attenuated the transcriptional level of fibronectin (Fig. 4b), a glycoprotein that functions as a scaffold for the ECM network facilitating cell proliferation and migration [33]. These results were further supported by assessing the expression profiles of matrix metalloproteinases (MMPs) and the tissue inhibitors of MMPs (TIMPs), which are essential secreted proteins known to maintain ECM turnover and homeostasis [34]. As shown in Fig. 4c, sorafenib raised the ratio of *MMP-2/TIMP-1* and *MMP-13/TIMP-1*, which indicates a net destruction of ECM by sorafenib in KFs (*p* < 0.05). Furthermore, sorafenib elicited a decreased expression of pro-fibrotic genes, such as *connective tissue growth factor* (*CTGF*), *interleukin-6* (*IL-6*), and *interleukin-8* (*IL-8*) (Fig. 4d). Similarly, the expression levels of antifibrotic genes *TGF-β3* and *IFN-γ* were enhanced by approximately 50 and 80 %, respectively, after treatment with sorafenib (Fig. 4e). On the other hand, sorafenib downregulated the expression levels of *IL-6* and *IL-8* but exhibited negligible effects on *TNF-α* and *IFN-γ* transcripts in normal fibroblasts (NFs) derived from human foreskins (Supplementary Fig. S2). Collectively, these results demonstrated that sorafenib exerts an antifibrotic role in KFs.

## Sorafenib eliminated cell migration and the invasion of KFs

**Fig. 5:**
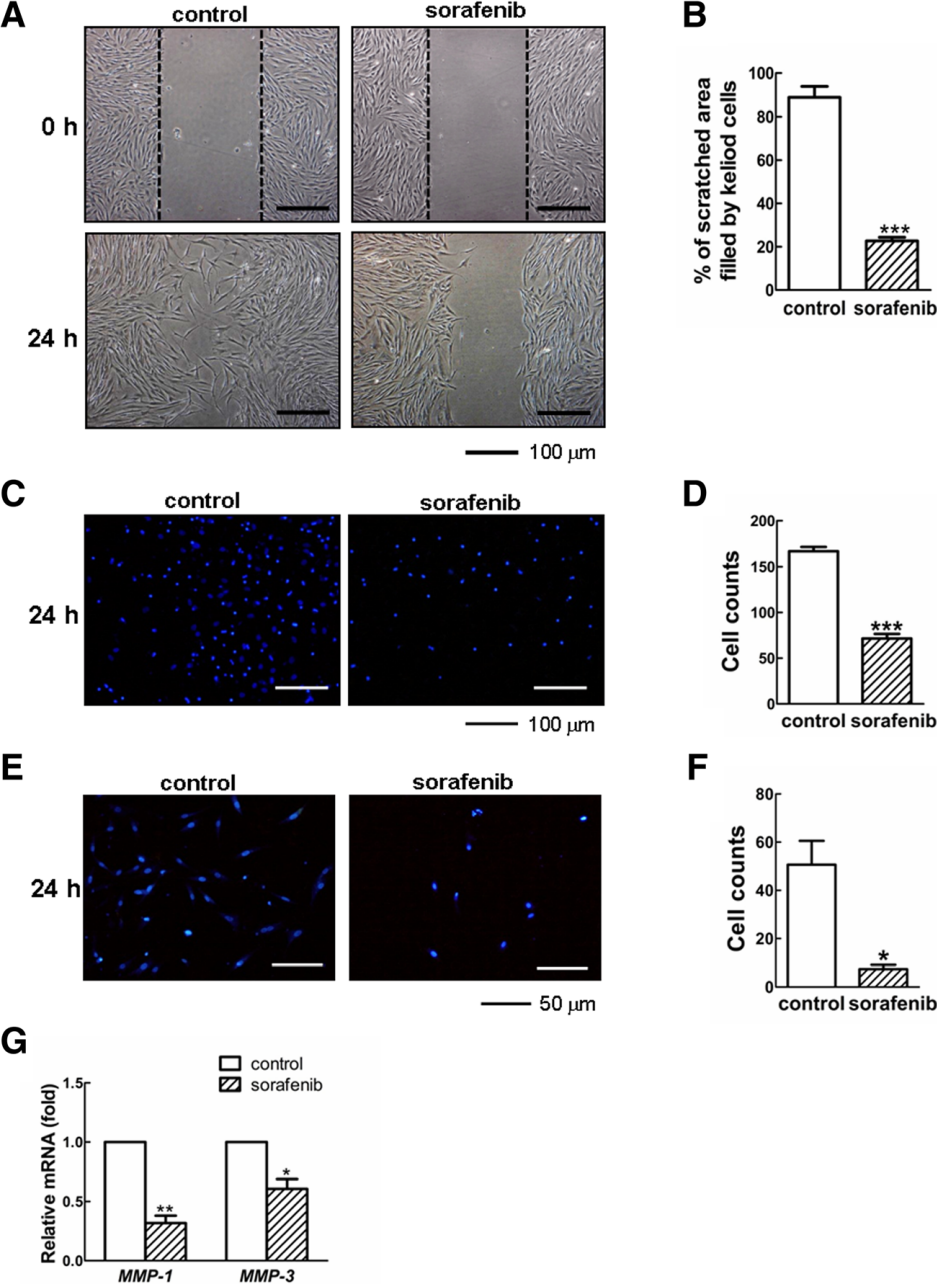
Sorafenib depresses the migratory and invasive abilities of KFs. **a**, **b** The KFs were grown to a confluent monolayer and treated with mitomycin C (10 μg/ml) for 2 h to inhibit cell proliferation. The confluent monolayer was wounded by manually scratching with a sterile pipette tip and following further incubation with or without sorafenib (5 μM) for 24 h. The scratched areas filled by migrated KFs were observed at 24 h post-scratching and quantified using IPP software. **c**, **d** As determined by transwell assay, the migratory KFs were visualized by imaging the nuclei labeled with DAPI. The number of migrated cells was counted in five randomly selected fields. **e**, **f** KFs were seeded in the upper chamber coated with Matrigel and incubated in the absence or presence of sorafenib (5 μM) for 24 h. The invasive KFs were visualized by imaging the DAPI-labeled nuclei and counted in five random fields. **g** The effects of sorafenib on the transcriptional levels of *MMP-1* and *MMP-3*. All the experiments were repeated in three independent cell pools (*n* = 3, 9 samples)

Because the enhanced migration and invasion properties of dermal fibroblasts are the critical parameters in the development of keloid disease [8, 32], we wondered if sorafenib would affect the cell behavior of KFs in vitro. As shown in Fig. 5a, KFs in the control group efficiently migrated into the scratched area to such an extent that the wound boundary was not apparent after 24 h of culture. In contrast, the migration capacity of KFs was evidently abrogated, as only 22.65 ± 3.33 % of the scratched area was filled after treatment with sorafenib (*p* < 0.001; Fig. 5b). These data were further validated by a transwell assay. As observed in Fig. 5c, sorafenib could strikingly reduce the number of cells that migrated across the filter membrane to the bottom surface. Compared with the nontreatment group (average 167 ± 7, *n* = 3), sorafenib led to a noticeable reduction in the number of migrated cells to approximately 43 % (average 72 ± 7, *n* = 3) (*p* < 0.001; Fig. 5d).

Meanwhile, the impact of sorafenib on cell invasion was assessed by Transwell invasion assay. As shown in Fig. 5e, control KFs had successfully invaded through the Matrigel and across the filter membrane after 24 h of incubation, whereas sorafenib-treated cells showed a greatly decreased invasive capability. Quantitative analysis by manual cell counting revealed that the number of invaded cells in the sorafenib-treated group was 87 % less than that in the control group (*p* < 0.05, Fig. 5f). Additionally, treatment with sorafenib largely blunted the transcriptional activities of *MMP1* and *MMP3* (*p* < 0.05, Fig. 5g), which are required to mediate cell invasion during wound repair and even tumor metastasis [35, 36]. Therefore, sorafenib appears to counteract the elevated capacities of migration and invasion in KFs.

## Sorafenib suppressed KF migration and proliferation in cultured keloid explants

**Fig. 6:**
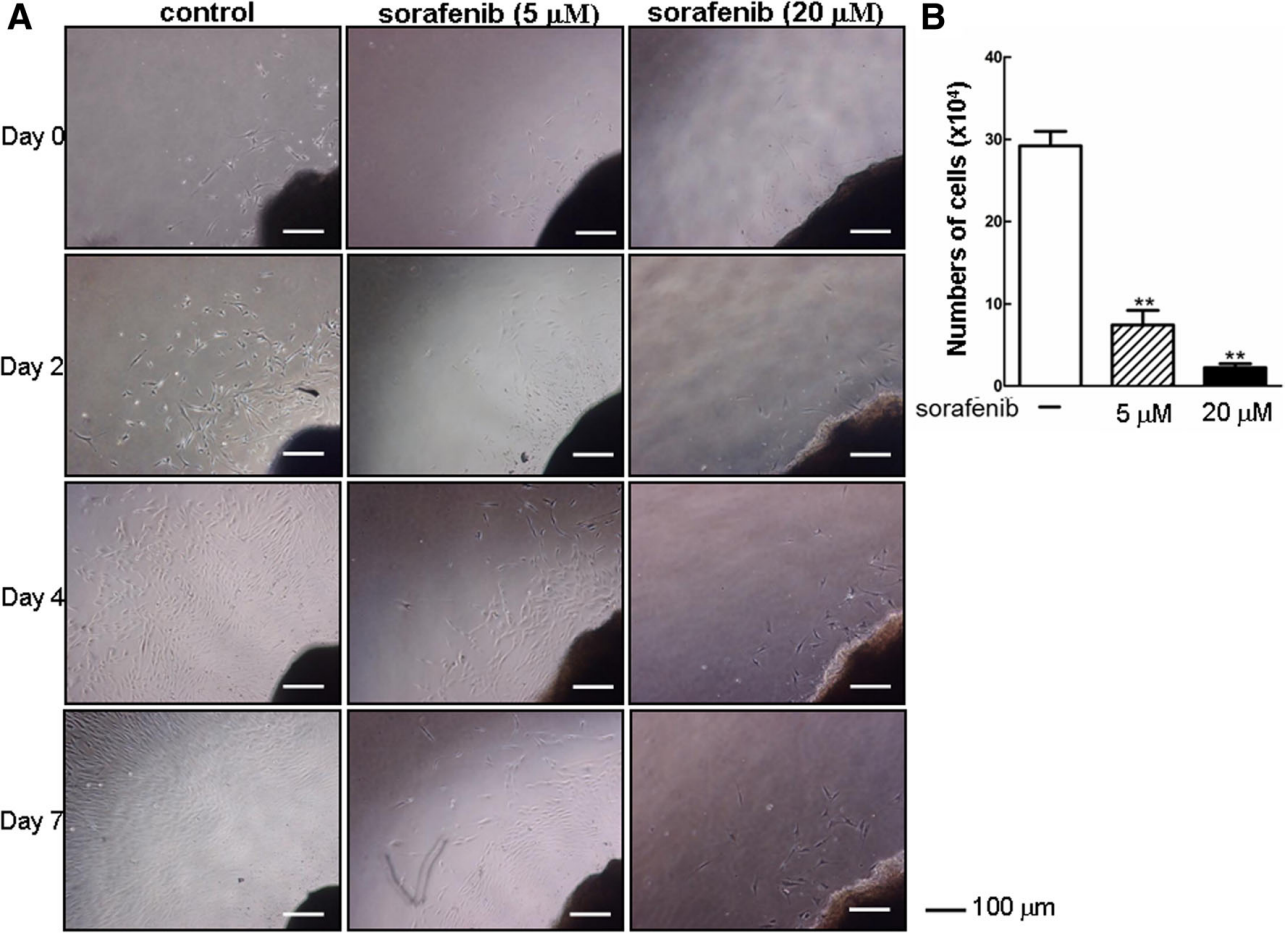
Sorafenib inhibits the migration and proliferation of cultured keloid explant-derived cells. **a** Three-millimeter keloid tissue explants were maintained in medium for 3 days and then treated with increasing concentrations of sorafenib (0, 5, and 20 μM) for additional days as indicated. Representative micrographs are shown in the same location where KFs were scratched at different time points as indicated. **b** The migrated KFs were collected at day 7 and counted using a hematocytometer. The experiment was repeated using four independent keloid tissue samples from different patients (*n* = 4)

The in vitro results outlined above encouraged us to further evaluate the therapeutic potential of sorafenib in keloid disease. In the absence of suitable animal models, we here applied an ex vivo keloid explant culture that represents a feasible clinically relevant model for studying keloid pathobiology as well as preclinical testing of potential therapeutics [23, 24]. In the vehicle group, spindle-like KFs initially migrated out from the edges of the explanted tissue and extended well into the Petri dish over a week. In contrast, treatment with sorafenib for 7 days induced a progressive decline in the number of KFs to approximately 74–95 % at day 7 (Fig. 6a, b), which may be due to a combination of its negative roles in cell migration and proliferation.

## Sorafenib depleted CD31^+^/CD34^+^ vessels and reduced collagen accumulation in keloid explant culture

**Fig. 7:**
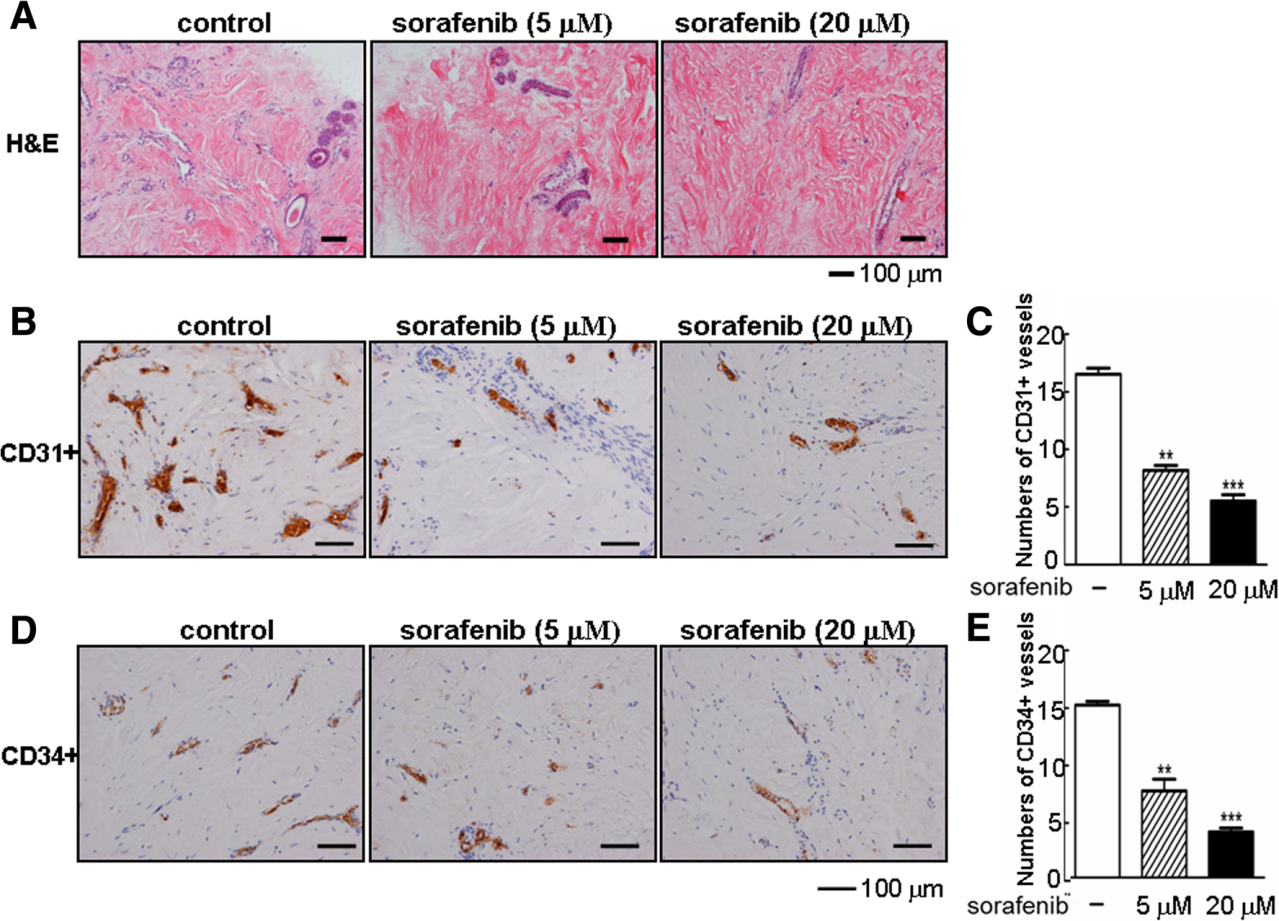
Sorafenib depletes angiogenesis in keloid explant culture. **a** After being cultured with or without sorafenib for 7 days, the keloid explants from different individuals (*n* = 3) were sectioned and stained with H&E for histological analysis. **b**, **e** The sections as indicated in the figures were also subjected to immunohistochemical analysis using antibodies against CD31 and CD34. Brown coloration indicates positive staining of related markers. The numbers of the CD31-positive endothelial cells and CD34-positive microvascular endothelial cells were counted in six randomly selected fields for each sample under a microscope

Histologically, keloids contain an increased blood vessel density compared with normal dermis or normal scars [37]. Hence, we wondered whether sorafenib would repress angiogenic activity. To better mimic the in vivo response to sorafenib, human keloid explants were embedded and sectioned for further analyses. As expected, we observed reduced cellularity and microvasculature in sorafenib-treated tissue sections (Fig. 7a). Interestingly, treatment with sorafenib at the higher concentration of 20 μM for 7 days caused a drastic decrease in the CD31-positive density, a traditional marker for endothelial cells (Fig. 7b). Quantification data revealed that approximately 65 % of CD31^+^ cells were reduced by sorafenib (Fig. 7c). Meanwhile, the microvascular endothelial cells were noticeably declined to 75 % in the papillary and reticular dermis, as visualized by CD34 immunostaining (Fig. 7d, e).

**Fig. 8:**
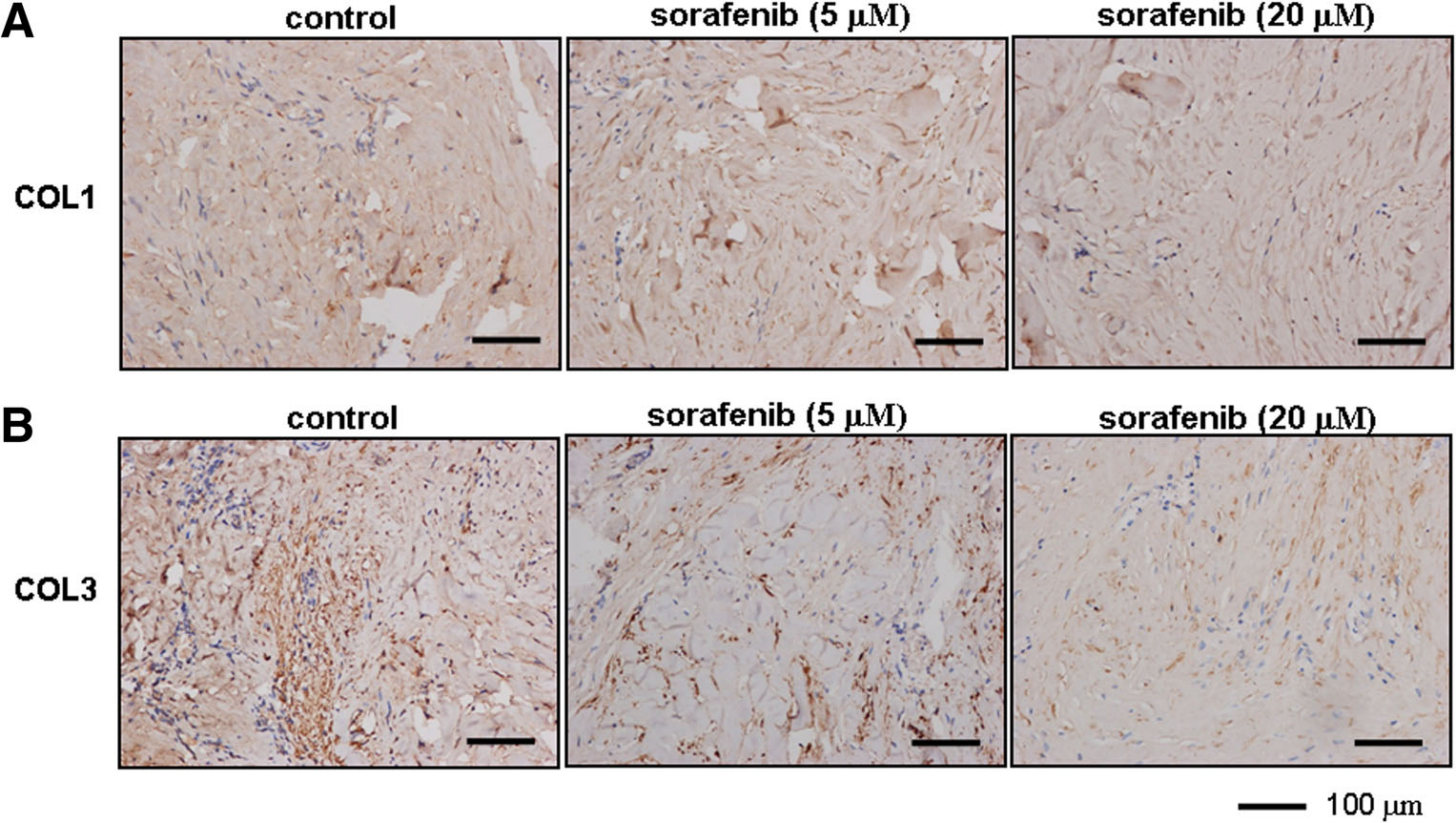
Sorafenib reduces collagen accumulation in keloid explant culture. **a**, **b** As described in Fig. 7, the keloid explants from different individuals (*n* = 3) were sectioned and subjected to immunohistochemical analysis. The immunoreactivity of collagen I (*COL1*) and collagen III (*COL3*) was tested to visualize collagen biosynthesis and deposition. Brown coloration indicates positive reactivity

Finally, we elucidated the potential anti-fibrotic effects of sorafenib in cultured keloid explants in situ. Compared with the vehicle group, treatment with sorafenib for 7 days reduced the immunoreactivity of collagen I (Fig. 8a), which is in accordance with the Western blotting and real-time qPCR results in KFs (Fig. 4a, b). Similarly, the accumulation of collagen III in human keloid explants was noticeably diminished by sorafenib even at a lower concentration (Fig. 8b). Collectively, these data suggested that sorafenib exerts its anti-angiogenic and anti-fibrotic properties in an ex vivo keloid model.

## Discussion

Keloid disease is an extremely complex disorder principally of the human dermis with notorious recurrence after various therapies used alone or in combination, leading to the concept that a keloid is a benign skin tumor [8, 17]. However, differing entirely from other tumors, keloids have only been observed in humans and have never been successfully reproduced in any animal models [25], probably reflecting our limited understanding of their etiology. Because human KFs are main inductive cells that initially show a marked infiltration in lesion tissue and subsequently mediate elevated ECM deposition [1, 9], innovative strategies that focus on targeting KFs by novel compounds may have important theoretical and practical implications for the clinical management of keloids. In the present study, we identified a novel property of sorafenib to antagonize intracellular signaling, and consequently, to substantially inhibit cell proliferation, migration, invasion, and ECM deposition using an in vitro cellular model (Figs. 2, 3, 4, and 5). Moreover, its anti-keloid efficacy was demonstrated in human cultured keloid explants, whereby sorafenib impeded KF migration and proliferation as well as angiogenesis and collagen accumulation (Figs. 6, 7, and 8). Encouraged by these findings, considerable clinical improvement and therapeutic value would be expected to cure keloids or improve their appearance. Therefore, we firmly believe that this chemical may have a much broader hands-on role than it is currently known.

It is increasingly recognized that MMPs are the major proteinases responsible for ECM degradation and remodeling [38]. An imbalance in the expression of MMPs and their inhibitors (TIMPs) is associated with the pathological process of dermal fibrosis in keloids [34, 35]. Specifically, MMP-2 and MMP-13 contribute extensively to break down types I and III collagens, the most abundant types of collagen in keloids and hypertrophic scars [32, 39]. Here, we observed that sorafenib not only reduced the expression of fibrotic ECM components (Figs. 4a, b and 8) but also modulated the *MMPs/TIMP-1* ratio to potentially promote the degradation of ECM proteins (Fig. 4c), thereby promising a reverse of the established fibrosis in keloids. Additionally, several members of the MMP family were reported to increase the migratory activity of KFs and tumor cells by degrading the basement membrane [1, 40]. For instance, both MMP1 and MMP3 actively participate in the ECM degradation of fibrotic scar tissue. However, it still remains controversial whether the expression of these two MMP enzymes would be elevated in keloids. The majority of literatures reported the upregulation of *MMP1* and *MMP3* [32, 39, 41], because they greatly contributed to the invasion of KFs, allowing the keloids to extend beyond the wound boundary. We speculated that the different stages of keloid development may attribute to the differential expressions of *MMPs*. The keloid specimens we harvested in this study were in a very active stage with aggressive invasion in our clinical observation, which may lead to an enhanced expression of them. Thus, *MMP-1* and *MMP-3* transcripts were apparently decreased after treatment with sorafenib (Fig. 5g), providing a possible explanation for its potent roles on controlling KF migration as well as tumor cell invasion and metastasis.

To fully elucidate the underlying mechanism of keloid pathogenesis and recurrence, it is essential to appreciate how normal skin repair is regulated and how this process goes awry. Normal wound healing is tightly controlled by a complicated network of distinct profibrotic and antifibrotic cytokines [1, 9]. With increased knowledge of this disease, TGF-β/Smad signaling has long been considered a pivotal fibrogenic inducer and therefore an important pharmacological target for treating keloids. In the past decade, several antifibrotic strategies have been successfully established based on the blockade or elimination of latent TGF-β signaling at various transduction steps. For example, neutralizing antibodies against TGF-β have been shown to prevent increased ECM deposition and reduce the rate of collagen synthesis in keloids and hypertrophic scars [13, 42, 43]. Similarly, the injection of TGF-β antibodies into the margins of healing dermal wounds in adult rats was shown to neutralize TGF-β signaling and thereby repair wound healing to reduce scar-tissue formation [44]. In addition to these protein-based therapies, small molecules and biological agents aimed at blocking this signaling cascade will provide an appropriate strategy for curing keloids. However, until now, widely effective therapeutic interventions for keloids have been currently lacking. The present study clearly showed that sorafenib counteracted TGF-β signaling by repressing the levels of both TGF-β1 expression and intracellular Smad2/3 phosphorylation (Fig. 2), suggesting that the sorafenib-mediated inhibition of cell proliferation, migration, and fibrotic matrix deposition is at least partly due to its interference with TGF-β/Smad signaling. In addition to TGF-β signaling, a growing body of growth factors has been reported to be dysregulated in keloid pathogenesis and recurrence [1, 9]. Here, we observed the inhibitory effects of sorafenib on the expression of profibrotic genes *VEGF*, *CTGF/CCN2*, *IL-6*, and *IL-8* (Figs. 2c and 4d), together with simultaneously synergistic effects on the expression of the antifibrotic genes *TGF-β3* and *IFN-γ* (Fig. 4e). Indeed, further definition of the exact nature of the crosstalk among cellular signaling pathways may provide a progressively clearer understanding of the molecular mechanisms underlying keloid pathogenesis and lead to new approaches for future therapeutic improvements.

Over the past decades, tremendous basic research efforts have been made to investigate the roles of receptor tyrosine kinases that are currently known to be key regulators of proliferative, inflammatory, and fibrotic diseases of the dermis [45]. Tyrosine-kinase inhibitors (TKIs), which target ligand-mediated receptors and intracellular signaling pathway activation, have a better opportunity for efficacy in the clinical treatments of these disorders, including keloids. As one of the most well-known TKIs, sorafenib blocks both Raf and a series of tyrosine kinases, such as PDGFR and VEGFR [16], suggesting a number of advantages over traditional approaches. Most importantly, the safety and tolerability of sorafenib have been well documented as it has been an FDA-approved agent for nearly a decade [14]. Additionally, the administration of sorafenib through topical injection or sustained systemic drug delivery to target specific diathesis may have significant clinical benefits. It is anticipated that systemic side effects known for sorafenib will not occur, if local administration is given at a low dose. As a matter of fact, an independent clinical trial is ongoing to assess the efficacy of sorafenib in patients with extensive keloids (see at https://clinicaltrials.gov/). In addition to sorafenib, the anti-keloid effectiveness of several potent TKIs has already been observed [46–48]. Despite improved insights into this therapeutic avenue, uncertainty and limitations remain in the translational research from the literature into its clinical practice. Certainly, more detailed investigations will be performed to warrant the potential application of sorafenib in the future.

In summary, we showed that the blockade of TGF-β/Smad and MAPK/ERK signaling pathways by sorafenib can effectively inhibit the proliferation, migration, cellular invasion, and ECM accumulation of KFs and concomitantly reduce the expression of inflammatory cytokines and inhibit angiogenesis, suggesting sorafenib as an attractive therapeutic intervention in the clinical setting of keloid disorder.

## Electronic supplementary material

Below is the link to the electronic supplementary material.

ESM 1 (PDF 597 KB)
