# Supplementary material for: Sorafenib exerts an anti-keloid activity by antagonizing TGF-β/Smad and MAPK/ERK signaling pathways
Source: J Mol Med (Berl). 2016 Jun 24;94(10):1181–94. doi: 10.1007/s00109-016-1430-3 (PMC5052317; doi:10.1007/s00109-016-1430-3)
Supplement: Supplementary file 1 — (PDF 597 KB) [file 109_2016_1430_MOESM1_ESM.pdf]

ELECTRONIC SUPPLEMENTARY MATERIAL

Sorafenib exerts an Anti-Keloid Activity by Antagonizing TGF-β/Smad and MAPK/ERK Signaling Pathways

Wenbo Wang[1,4], Miao Qu[1,4], Lan Xu[2,4], Xiaoli Wu[1], Zhen Gao[1], Tingyu Gu[2], Wenjie Zhang[1,3], Xiaoyan Ding[2], Wei Liu[1,3]* and Yue-Lei Chen[2]*

Supplementary Figure S1 The low concentrations of DMSO were nontoxic to KFs. (A) The cell viabilities of KFs treated with low concentrations of DMSO (0.05% and 0.2%) were measured with a CCK-8 assay at days 1, 3, 5 and 7. A negligible effect of DMSO on cell viability was observed as compared to non-treated control. (B-C) After treatment with 0.05% DMSO (v/v) for 48 h, KFs were subjected to real-time qPCR to detect the genotoxicity of DMSO on the transcripts of ECM molecules, pro-fibrotic and antifibrotic cytokines. KFs in the growth medium were used as a non-treated control. (D-E) KFs were pretreated with mitomycin C (10 μg/ml) for 2 h to block cell proliferation. Then the cell monolayer was scratched and incubated in the culture medium with or without vehicle DMSO (0.05%). The scratched areas filled by migrated KFs were observed at 24 h post-scratching and quantified using IPP software.

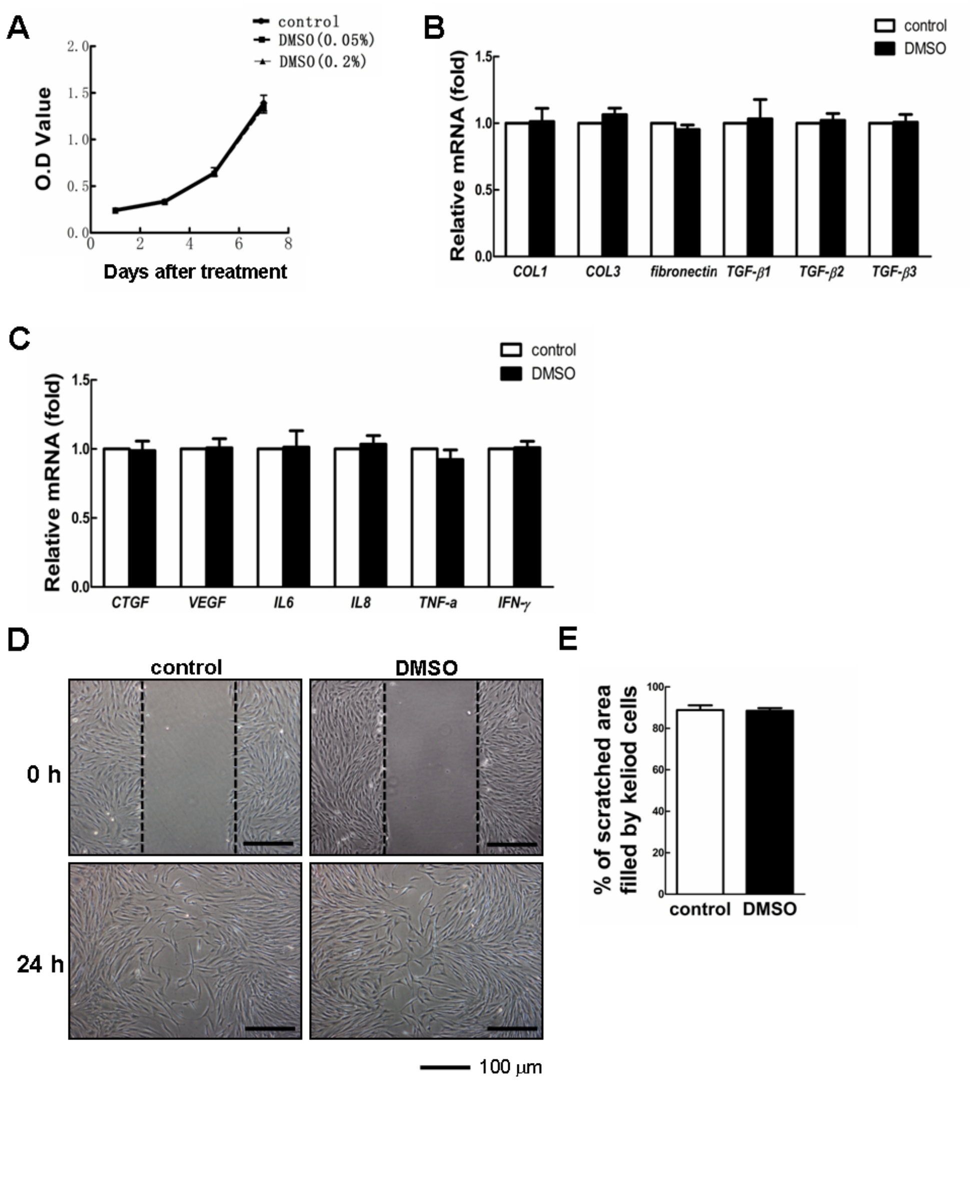

Supplementary Figure S2 Sorafenib reduces the expression levels of two pro-inflammatory genes in NFs derived from human foreskins. After treatment with sorafenib (5 μM) for 48 h, NFs were subjected to real-time qPCR to detect the effects of sorafenib on the expression levels of pro-inflammatory genes including *IL-6*, *IL-8*, *TNF-α* and *IFN-γ*. DMSO was used as solvent control. The qPCR assay was performed in triplicate and repeated in three independent cell pools (n=3, 9 samples).

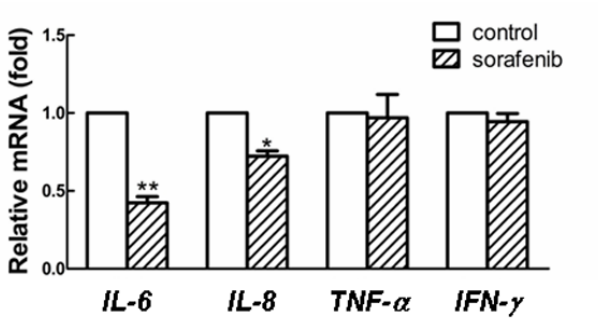

Supplementary Table S1. Demographic data of keloid samples used in this study.

Sample No.	Gender	Ethnic Background	Age	Site of Specimen Collected	The Size of the Specimens (cm^2)
**KS1**	M	Asian	35	Chest	8×0.5
**KS2**	M	Asian	41	Chest	5×3
**KS3**	M	Asian	28	Chest	3×2
**KS4**	M	Asian	28	Chest	5×6
**KS5**	F	Asian	31	Chest	10×2
**KS6**	M	Asian	32	Chest	7×3
**KS7**	M	Asian	23	Chest	3×2; 2×1
**KS8**	F	Asian	35	Chest	3×1.5
**KS9**	M	Asian	38	Chest	4.5×2
**KS10**	F	Asian	26	Chest	4×3
**KS11**	F	Asian	27	Chest	3.5×2
**KS12**	F	Asian	29	Chest	4×1; 4×3; 2×1
**KS13**	M	Asian	33	Chest	6×3
**KS14**	M	Asian	27	Chest	6×3
**KS15**	F	Asian	24	Chest	3×2
**KS16**	F	Asian	23	Chest	2×1
**KS17**	F	Asian	26	Chest	3.5×2.5
**KS18**	M	Asian	26	Chest	5×3
**KS19**	F	Asian	45	Chest	10×3
**KS20**	F	Asian	30	Chest	4×2; 2.5×2
**KS21**	F	Asian	55	Chest	5.5×3
**KS22**	F	Asian	35	Chest	10×2
**KS23**	M	Asian	26	Chest	3.5×3
**KS24**	F	Asian	24	Chest	5×4
**KS25**	F	Asian	25	Chest	7.5×5.5
**KS26**	F	Asian	26	Chest	6×5
**KS27**	F	Asian	30	Chest	3×2
**KS28**	M	Asian	20	Chest	11×1.5
**KS29**	F	Asian	27	Chest	5×3
**KS30**	M	Asian	49	Chest	3×1; 8×2; 2×1

Note: Samples KS27-KS30 were used for *ex-vivo* keloid explant culture.

Supplementary Table S2 Primers used in quantitative PCR analysis.

Gene Name	Primer Sequence (5'-3')	Product Size (bp)
Collagen I	Sense: GGCGGCCAGGGCTCCGACCC	347
	Antisense: AATTCCTGGTCTGGGGCACC	
Collagen III	Sense: TGGTGTTGGAGCCGCTGCCA	373
	Antisense: CTCAGCACTAGAATCTGTCC	
Fibronectin	Sense: GCCACTGGAGTCTTTACCACA	61
	Antisense: CCTCGGTGTTGTAAGGTGGA	
TGF-β1	Sense: AAGGACCTCGGCTGGAAGTG	136
	Antisense: CCGGGTTATGCTGGTTGTA	
TGF-β2	Sense: CAGCACACTCGATATGGACCA	113
	Antisense: CCTCGGGCTCAGGATAGTCT	
TGF-β3	Sense: GGTTTTCCGCTTCAATGTGT	119
	Antisense: GCTCGATCCTCTGCTCATTC	
SMAD7	Sense: CCTTAGCCGACTCTGCGAACTA	131/134
	Antisense: CCAGATAATTCGTTCCCCCTGT	
MMP1	Sense: GGAGCTGTAGATGTCCTTGGGGT	139
	Antisense: GCCACAACTGCCAAATGGGCTT	
MMP2	Sense: CAAAAACAAGAAGACATACATCTT	232
	Antisense: GCTTCCAAACTTCACGCTC	
MMP3	Sense: AGGACAAAGCAGGATCACAGTTG	68
	Antisense: CCTGGTACCCACGGAACCT	
MMP13	Sense: AGTGGTGGTGATGAAGATGATTTG	144
	Antisense: CATTTCTCGGAGCCTCTCAGTC	
TIMP1	Sense: TGACATCCGGTTCGTCTACA	102
	Antisense: TGCAGTTTTCCAGCAATGAG	
CTGF	Sense: ACAAGGGCCTCTTCTGTGACTT	102
	Antisense: GGTACACCGTACCACCGAAGAT	
VEGF	Sense: TGCAGTTATGCGGATCAAACC	80
	Antisense: TGCATTCACATTTGTTGTGCTGTA	
IL-6	Sense: CCTGACCCAACCACAAATGC	157
	Antisense: ATCTGAGGTGCCCATGCTAC	
IL-8	Sense: TCCTGATTTCTGCAGCTCTGTGTG	161
	Antisense: AATTTCTGTGTTGGCGCAGTGTGG	

TNF-α	Sense: CTCGAACCCCGAGTGACAAG	159
	Antisense: TGAGGTACAGGCCCTCTGAT	
IFN γ	Sense: TGCAGGTCATTCAGATGTAGC	269
	Antisense: GGACATTCAAGTCAGTTACCG	
HPRT1	Sense: GCCATCACATTGTAGCCCTCT	308
	Antisense: TGCGACCTTGACCATCTTTGG	
